# One-Week Home-Based HRV Biofeedback with Supervised Sessions Versus Passive Relaxation: Effects on Autonomic, Sensorimotor Functions and Kata Performance in Eastern Martial Arts Athletes

**DOI:** 10.3390/sports14020051

**Published:** 2026-02-03

**Authors:** Nikola Toloraya, Anastasia Kovaleva, Ivan Belousov, Albina Andreeva

**Affiliations:** 1 Federal Research Center for Innovator and Emerging Biomedical and Pharmaceutical Technologies, 125315 Moscow, Russia; kovaleva_av@academpharm.ru (A.K.); belousov_ia@academpharm.ru (I.B.); moymio@yandex.ru (A.A.); 2 Department of Physiology, Federal State Budget Educational Institution of Higher Education “The Russian University of Sport GTSOLIFK”, 105122 Moscow, Russia

**Keywords:** biofeedback (BFB), heart rate variability (HRV), resonant breathing, martial arts, athletes, sensorimotor functions, kata performance

## Abstract

Heart Rate Variability (HRV) biofeedback could be considered as a tool to help athletes to optimize their performance. This study aimed to examine the effects of a one-week HRV biofeedback (HRV-BFB) program on physiological indices, sensorimotor functions, and kata performance in Eastern martial arts athletes. Forty high-level martial arts athletes (karate, wushu, taekwondo, kyokushinkai) aged 17–27 years were divided into two groups: a control group (n = 20) and a biofeedback group (BFB, n = 20). Athletes from both groups underwent assessment of sensorimotor functions and the technical quality of their kata routines. The primary outcome was the expert-rated kata performance score. All routines were video-recorded and independently rated by three certified judges. The BFB group completed a hybrid HRV-BFB program consisting of supervised resonance-frequency breathing sessions in the laboratory and one week of home-based practice. During supervised sessions, athletes performed slow abdominal-paced breathing (6 breaths/min). At home, they practiced the same breathing pattern twice daily for one week (5 min per session, smartphone-guided). Nonparametric tests were used because several variables deviated from normality, and the sample size per group was limited (n = 20). After completing the HRV-BFB training, movement oscillation frequency improved significantly, reflected by lower movement oscillation frequency (*p* = 0.0009, r = 0.79), faster choice reaction time at a tendency level (*p* = 0.0793, r = 0.39), and an increase in blood volume pulse (BVP) (*p* = 0.037, r = 0.48) in BFB group compared to control group. Following BFB training, the judges’ scores did not change in the control group, while a significant increase was observed in the BFB group (*p* = 0.038, r = 0.44), indicating a positive effect of BFB training on kata performance. Regular HRV-BFB training emphasizing slow-paced abdominal breathing may enhance autonomic regulation, fine motor control, and improve the technical execution of kata routines in athletes.

## 1. Introduction

Martial arts is a combat discipline requiring an integration of physiological efficiency, precise motor control, and psychological endurance. Among its various disciplines, kata performance is especially demanding. It consists of choreographed sequences that emphasize precision, rhythm, and technical execution. Successful kata execution depends on optimal regulation of autonomic function, fine motor coordination, and emotional control [1,2].

Because autonomic regulation plays a central role in kata performance, recent research has increasingly examined HRV biofeedback as a practical approach to improve psychophysiological self-regulation. HRV reflects the variation in intervals between heartbeats and is widely accepted as an index of autonomic nervous system activity, particularly parasympathetic modulation. Higher HRV is associated with greater physiological adaptability, cardiovascular fitness, and better executive functioning [3,4].

HRV biofeedback (HRV-BFB) is based on paced breathing at an individual’s resonance frequency (approximately 0.1 Hz, 6 breaths per minute), a strategy shown to support autonomic balance through vagally mediated mechanisms, including enhanced baroreflex function and improved self-regulatory capacity [5,6]. The method has demonstrated ecological validity, with applications extending to athletic training environments using wearable devices [7].

Empirical studies have demonstrated that HRV-BFB may benefit athletes in sports requiring precise timing and motor control, such as soccer, archery, and basketball, by supporting reaction time, movement consistency, and attentional focus [8,9,10]. These findings suggest that HRV-BFB may be particularly relevant for disciplines characterized by high psychophysiological demands and fine motor precision.

Despite its demonstrated benefits across various sports, HRV-BFB remains underexplored in martial arts, particularly in kata performance. The internal pacing, motor accuracy, and psychophysiological demands of kata make it a compelling model for studying HRV-BFB effects. Preliminary findings indicate that HRV-BFB may support performance under stress by modulating autonomic balance and sensorimotor control [5,11].

Additional benefits of HRV-BFB include improved sleep quality, anxiety regulation, and physiological recovery; these factors are highly relevant for athletes managing competitive stress and intensive training loads [12,13,14]. Conceptual frameworks such as the neurovisceral integration model and polyvagal theory further support the role of vagally mediated HRV in supporting self-regulation during complex motor tasks [15].

This study aimed to evaluate the short-term associations of HRV-BFB with autonomic regulation, sensorimotor function, and kata performance in eastern martial artists. This study was primarily performance-oriented, with mechanistic inferences limited to autonomic and sensorimotor markers.

## 2. Materials and Methods

This study was conducted at the Department of Physiology, Federal State Budget Educational Institution of Higher Education “The Russian University of Sport (GTSOLIFK)” between 2024 and 2025 (during the early preseason (weeks 2–4)). All tests took place during preseason training sessions and were administered by two faculty members of the Department of Physiology. Participants had no musculoskeletal injuries or contraindications to physical testing. Written informed consent was obtained from all subjects prior to participation.

The study protocol was approved by the Local Bioethics Committee of GTSOLIFK (Protocol No. 15, 9 September 2024) and was conducted in accordance with the proncoples of the 2013 revision of the Helsinki [16] World Medical Association. World Medical Association Declaration of Helsinki: Ethical Principles for Medical Research Involving Human Subjects. JAMA. 2013;310(20):2191–2194. doi:10.1001/jama.2013.281053.

All assessments were conducted by two trained research assistants with prior experience using the psychophysiological measurement systems. Both assessors were blinded to participant group allocation throughout pre- and post-intervention testing. All assistants and judges did not have access to the assignment of athletes to groups. Kata judges evaluated videos anonymously and were not informed about group identity.

Experimental design

This study used a parallel-group design: an HRV-BFB intervention group and a time-matched control group that watched relaxation videos. Outcome assessments included: heart rhythm and respiratory parameters (baseline physiology), sensorimotor functions (reaction time, movement oscillation frequency, object tracking, force differentiation), and expert-rated kata performance. By integrating both physiological and behavioral measures, this study aims to strengthen the empirical evidence supporting HRV-BFB as a practical training tool in combat sports. Randomization was stratified by martial art discipline to ensure proportional distribution.

Participants

A total of 40 competitive high-level athletes (karate, wushu, taekwondo, kiokyshinkai) participated in this study (Table 1). All participants were experienced in kata performance and had at least 10 years of formal training. Inclusion criteria were: (1) active training status; (2) no history of cardiovascular, respiratory, neurological, or psychiatric disorders; and (3) not currently receiving psychophysiological or breathing-based interventions. Exclusion criteria were: (1) prior biofeedback or meditation experience, (2) respiratory disorders affecting paced breathing, (3) acute injuries, (4) use of any medications that could affect cardiovascular or autonomic regulation.

A formal a priori power analysis was not conducted; however, our sample size (n = 40) is consistent with sample sizes in similar HRV-BFB studies on athletic performance, which typically include 20–36 participants and report medium effect sizes.

Athletes were randomly assigned to one of two experimental conditions: the HRV biofeedback group (BFB group) or the control group. Participants completed a baseline questionnaire assessing demographics, recent sleep duration, and athletic experience. A second questionnaire assessed pre-competitive sport-related anxiety, based on self-report prior to the intervention [16].

According to the pre-intervention questionnaire, athletes in the BFB and control groups did not differ in the number of hours they trained per week prior to testing (*p* = 0.085). Although participants represented several martial arts disciplines, the general structure, content, and intensity of their regular training routines were comparable between groups, allowing us to consider the overall training load as similar.

Across martial arts disciplines that include kata routines (Table 2), execution time ranged from 60 to 360 s depending on discipline rules. Each second exceeding or not exceeding the prescribed duration is penalized according to the rules of each specific sport.

Tests and assessments

Sensorimotor and Physiological Assessments

Before and after the intervention period, all participants underwent a standardized assessment battery. All sensorimotor tests were performed using certified neurophysiological systems NS PsychoTest (Neurosoft LLC, Ivanovo, Russia; software version 1.6.9.3), available at: https://neurosoft.com (accessed on 28 January 2026). routinely applied in sports psychophysiology. These computerized sensorimotor tasks have established reliability and validity, with published test–retest indices summarized in Supplementary Table S1 [17,18,19,20,21,22].

Simple visual–motor reaction time: In this task, participants were presented with a visual stimulus (green light) at random time intervals. They were instructed to respond as quickly as possible by pressing a button upon the appearance of the light. The latency between stimulus onset and the participant’s response was recorded as the simple visual–motor reaction time.

Choice reaction time: In this task, participants were presented with a sequence of visual stimuli in a randomized order. The sequence included target stimuli, to which participants were instructed to respond by pressing a button on a handheld response tube, and distractor stimuli (neutral signals), which required no response. Both reaction time to the target stimuli and errors of omission (missed target) and commission (false response to distractor) were recorded in the database.

Coincidence Anticipation Timing Test (Reaction to a moving object): This task evaluated participants’ ability to respond to a dynamic visual stimulus. On the computer screen, two concentric circles were displayed, each marked with a “START” and “STOP” label. Beginning at the “START” mark, a green-colored segment—representing a moving “stream”—began to progress clockwise along the annular space between the circles. The participant was instructed to press a response button precisely when the moving segment passed through the “STOP” mark. The time interval between the exact passage of the stimulus and the participant’s response was recorded as the reaction latency.

Grip-force differentiation was assessed using a calibrated dynamometer with visual feedback: Athletes first applied 20 kg of force while observing a visual scale. Then, with eyes closed, they were instructed to produce 21–22 kg and 18–19 kg of force based on internal perception alone.

Stylus Tracking Task: Participants, while in a standing position, were asked to guide a stylus through a labyrinth pattern without touching its borders.

Baseline physiological recordings were conducted twice (pre- and post-intervention) under resting conditions (sitting position, 5 min duration, controlled breathing avoided, recording time standardized (10:00–14:00). All physiological recordings were conducted using the FlexComp Infiniti System (Thought Technology Ltd., Montreal, QC, Canada), a standard platform for psychophysiological monitoring in research and applied settings. HRV was measured using a photoplethysmographic (PPG). Respiratory rate and amplitude were monitored via a pneumograph sensor placed around the abdominal and thoracic regions. Resting beat-to-beat intervals were derived from photoplethysmography (PPG); sensor was attached to the left thumb. Previous studies have shown that pulse rate variability obtained from PPG provides a valid surrogate for ECG-derived HRV under resting conditions, particularly during seated measurements with minimal movement, where agreement between methods is highest. However, it is also acknowledged that PPG-based HRV may be more sensitive to motion and peripheral factors compared with ECG and should therefore be interpreted cautiously [23].

Physiological signal processing and biofeedback protocols were administered via BioGraph Infiniti Software (Thought Technology Ltd., Montreal, QC, Canada; version 6.0). HRV indices were derived from the PPG signal using Kubios HRV Standard software (version 3.5.0; Kubios Oy, Kuopio, Finland) including time-domain, frequency-domain, and nonlinear parameters. Respiratory data were processed using BioGraph built-in analysis tools.

Kata Performance Assessment

**Figure 1:**
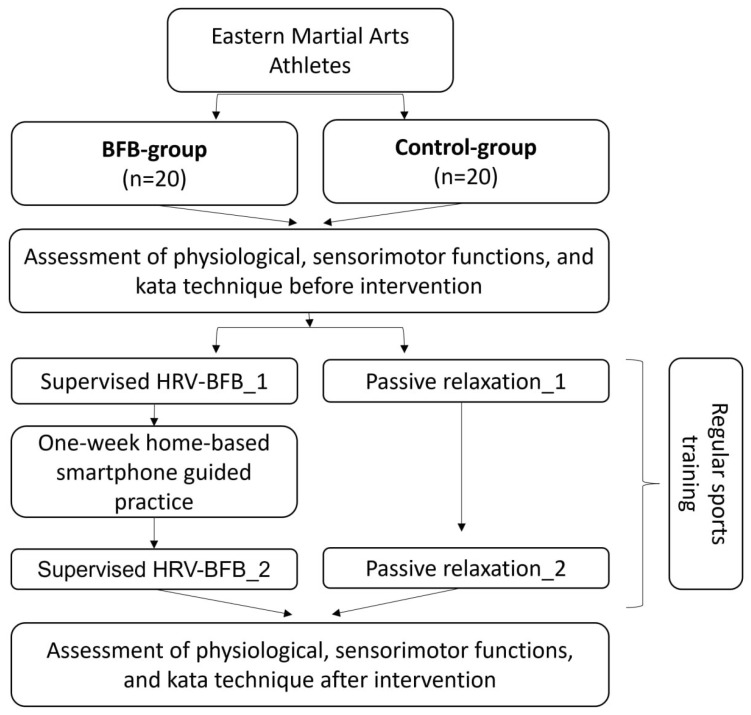
Flowchart of this study.

All athletes performed a kata sequence before and after the intervention. Performances were recorded and independently evaluated by a panel of three certified kata experts, each sport using standardized judging criteria (Figure 1).

**Intervention Procedures**

**BFB Group**

The HRV-BFB protocol consisted of a structured hybrid program combining supervised laboratory sessions with a one-week period of daily home practice. The intervention comprised four supervised sessions in the laboratory:

Session 1 (baseline visit): informed consent, questionnaire completion, baseline physiological recording, sensorimotor testing, and kata performance.

Session 2 (day 2): training in abdominal breathing techniques using the Abdominal & Thoracic Amplitude Training protocol Biograph Infiniti Software and determination of each participant’s resonance frequency using an adaptive paced-breathing protocol [24,25].

Session 3 (day 3): supervised HRV-BFB training at individually determined resonance frequency, emphasizing slow-paced abdominal breathing.

Session 4 (post-intervention visit): following the week of home practice, participants completed a repeated resonance-frequency assessment, a supervised HRV-BFB session, and post-intervention testing (sensorimotor assessments and kata performance).

Between supervised sessions 3 and 4, athletes completed one week of daily home-based breathing practice, consisting of two 5 min sessions per day guided by a smartphone breathing pacer. Adherence to home practice was monitored via daily self-report, screenshots of each completed session, and reinforced at the final laboratory visit.

**Control Group**

Participants in the control group visited the laboratory at the same frequency and duration. Control participants watched standardized relaxation videos, while physiological signals were recorded under identical conditions.

**Statistics**

The statistical analysis was performed using the software Statistica v.12 (StatSoft, Inc., Tulsa, OK, USA). Nonparametric methods were used due to the limited sample size (20 people in each group). The comparison of indicators before and after intervention within the same group of athletes was carried out using the Wilcoxon criterion for related samples (Wilcoxon Matched Pairs Test). The Mann–Whitney U Test, designed to analyze independent samples, was used to assess the intergroup differences at the stages before and after the intervention. Effect sizes for nonparametric comparisons were calculated as r = Z/√N, where N is the total sample size.

Given the exploratory nature of this study and the limited sample size, no formal correction for multiple comparisons was applied; results are therefore interpreted cautiously in conjunction with effect size estimates.

The unit of analysis is the individual athlete. Physiological and sensorimotor data were directly measured per participant. For kata performance, the three expert ratings for each athlete’s routine were aggregated using a standard methodological approach: the mean of the three judges’ scores was calculated for each athlete. This created a single performance variable at the participant level, ensuring that our between-group comparisons are based on the defined unit of analysis. While we acknowledge that reporting inter-rater reliability would be an additional strength, the use of multiple certified judges and the standard practice of averaging their scores mitigates the potential impact of individual rater bias on the group-level results.

Given the exploratory nature of this study and the limited sample size, no formal correction for multiple comparisons was applied. Instead, results are interpreted cautiously, with emphasis placed on consistency of effects and effect size estimates rather than on isolated *p*-values.

## 3. Results

In the control group, several HRV indices (VLF%, LF%, HF%) (Table 3) increased significantly after the intervention: VLF% (*p* = 0.0438), LF% (*p* = 0.0169), HF% (*p* = 0.008), LF and HF n.u. (*p* = 0.009), LF/HF ratio (*p* = 0.0333).

**Figure 2:**
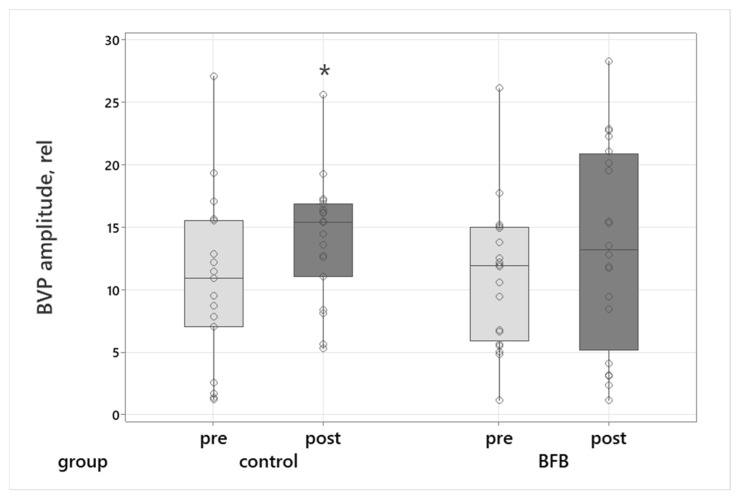
BVP amplitude mean (rel) before (pre) and after (post) the intervention in the Control and BFB groups. Box plots show median, interquartile range, and whiskers (min-max). Circles represent individual participant’s values. “*”—indicates a statistically significant pre-post difference.

In the control group, BVP amplitude showed a small but statistically significant increase (Wilcoxon test: Z = 2.09, *p* = 0.037), with a medium effect size (r = 0.48) (Figure 2).

**Figure 3:**
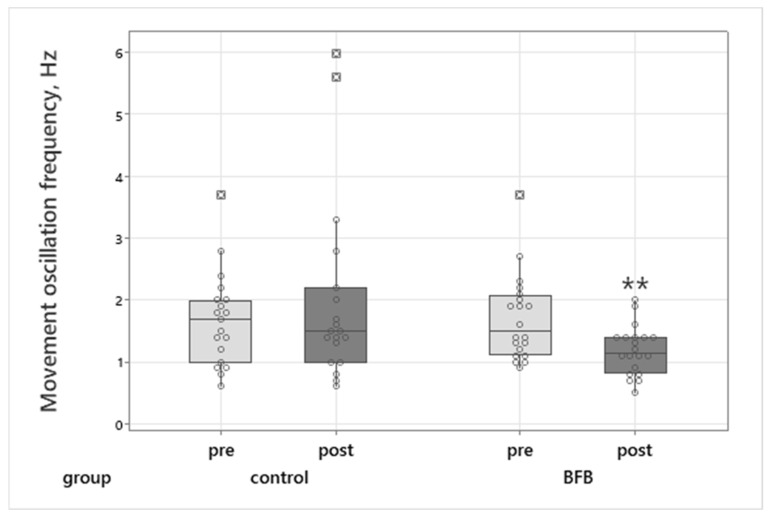
Movement oscillation frequency (Hz) in BFB and control groups before (pre) and after (post) intervention or relaxation. Box plots show median, interquartile range, and whiskers (min-max). Circles represent individual participant’s values. “**”—indicates a statistically significant pre-post difference.

Movement oscillation frequency significantly decreased in the BFB group (Wilcoxon test: Z = 3.44, *p* = 0.0006), with a very large effect size (r = 0.79) (Figure 3).

**Figure 4:**
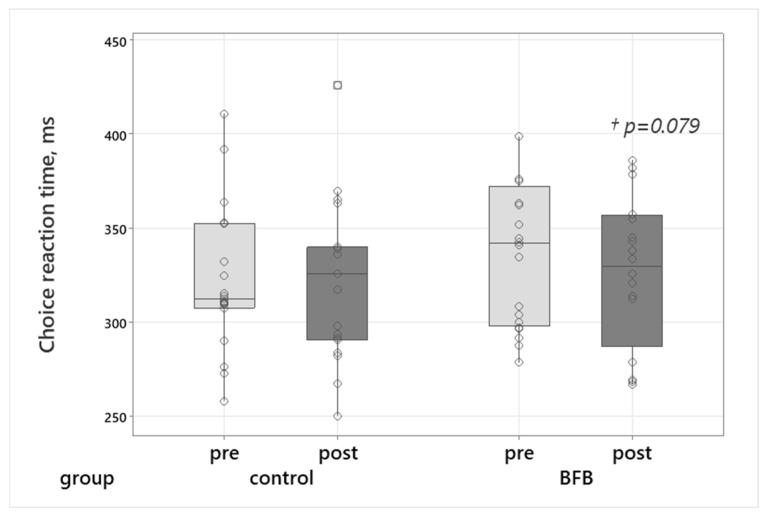
Choice reaction time (ms) before and after the intervention in the control and BFB groups. Box plots show median, interquartile range, and whiskers (min-max). Circles represent individual participant’s values.

A trend toward faster choice reaction time was also observed in the BFB group (Z = 1.76, *p* = 0.0793, r = 0.39 (medium effect)) (Figure 4).

**Figure 5:**
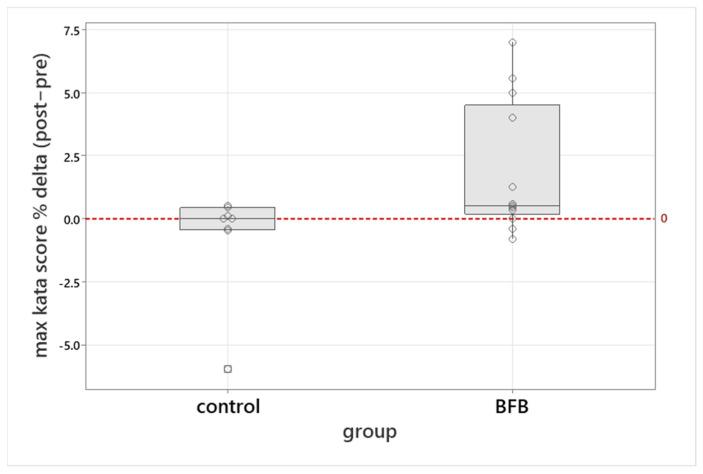
Change in judges’ scores (% delta) in kata after BFB intervention and in the control group. The data is presented as a percentage of difference from the maximum score (after—before) for the kata. The red dotted line indicates no change in the judges’ scores. Box plots show median, interquartile range, and whiskers (min-max). Circles represent individual participant’s values.

Because different martial arts disciplines use different maximum scoring limits, all judging outcomes were converted to percentages representing the proportion of the maximal possible score in the respective discipline (e.g., karate vs. taekwondo). The three evaluations were then averaged to produce a single performance index for each athlete. This index remained unchanged in the control group but increased significantly after training in the BFB group (U = 27.0, Z = 2.07, *p* = 0.038), with a medium-to-large effect size (r = 0.44), indicating a positive effect of HRV-BFB on kata execution (Figure 5).

Overall, the between-group comparisons demonstrated that HRV-BFB was associated with improvements in two key sensorimotor outcomes—reduced movement oscillation frequency and a trend toward faster choice reaction time—while no such changes were observed in the control group. In contrast, the control group showed significant alterations in several HRV spectral indices that were not present in the BFB group. Additionally, expert-rated kata performance increased only in the BFB group, whereas scores remained unchanged in controls. These findings highlight distinct response patterns between the two groups across autonomic, sensorimotor, and performance-related measures.

## 4. Discussion

Given growing interest in HRV biofeedback as a tool for psychophysiological self-regulation, the present study examined its short-term associations with autonomic, sensorimotor, and performance-related outcomes in high-level Eastern martial arts athletes. Specifically, we compared changes in cardiovascular markers (including BVP), selected sensorimotor measures (movement oscillation frequency and reaction time), and expert-rated kata performance following a hybrid HRV-BFB protocol versus passive relaxation.

### 4.1. Physiological Parameters in Elite Martial Art Athletes

Passive relaxation was accompanied by measurable changes in cardiovascular and HRV indices, whereas the HRV-BFB group showed comparatively stable autonomic patterns across sessions. In the control group, pulse-wave amplitude increased, and shifts in HRV spectral composition were observed, including higher VLF% and LF%, lower HF%, and an elevated LF/HF ratio. Although these changes occurred in a relaxed context, similar patterns have been reported during low-demand or resting tasks and have been interpreted as reflecting vigilance or subtle cognitive–emotional engagement rather than unequivocal sympathetic activation [26,27]. In contrast, athletes in the BFB group did not exhibit significant pre-post changes in these parameters. This relative stability may be consistent with prior work showing that resonance-frequency breathing can support more consistent autonomic patterns over time [4], although our study did not directly assess underlying mechanisms such as baroreflex function or vagal modulation. Recent findings further indicate that HRV-BFB may contribute to longer-term autonomic resilience and improved emotion regulation rather than producing immediate shifts in resting HRV metrics [28,29]. Taken together, these observations suggest that passive relaxation was associated with short-term alterations in vascular and autonomic markers, while HRV-BFB practice appeared compatible with a more stable autonomic profile during repeated assessments.

### 4.2. Sensorimotor Parameters in Elite Martial Art Athletes

Significant improvements were observed in the biofeedback (BFB) group in two key indices of neuromotor control: reduced movement oscillation frequency and improved choice reaction time. These changes may reflect enhancements in neuromotor precision and cognitive–motor processing, both of which are relevant for tasks requiring steadiness, timing, and movement control. The reduction in movement oscillation frequency following HRV-BFB may be compatible with previous findings suggesting that slow-paced breathing is associated with reduced sympathetic activation and increased parasympathetic influences [24,30]. Earlier research indicates that resonance-frequency breathing can stabilize motor output and attenuate involuntary oscillations through autonomic pathways [4,31]. Such mechanisms, if present, could plausibly contribute to more consistent control of distal musculature during static or semi-static motor tasks.

A tendency toward improved choice reaction time may indicate facilitation of higher-order sensorimotor processing rather than simple neuromuscular acceleration. Unlike simple reaction time, choice tasks require selective attention, response selection, and inhibitory control—domains that have been shown to be sensitive to autonomic-cortical interactions [32,33]. Prior studies report that HRV-BFB may support prefrontal efficiency and attentional regulation [34,35], and the moderate reduction in reaction latency observed here may be consistent with these findings, although causal pathways cannot be inferred from the present data.

Other sensorimotor measures remained unchanged, which is consistent with the notion that elite athletes often perform near their functional ceiling, leaving limited room for short-term improvements [36,37,38]. Additionally, kata routines rely heavily on anticipatory control and rhythmic coordination rather than reactive motor adjustments, which may explain why improvements were more evident in cognitive–motor tasks than in purely sensory or force-based measures [39,40].

Taken together, the results suggest that short-term HRV-BFB may be associated with improvements in neuromotor steadiness and cognitive–motor integration in elite Eastern martial arts athletes while acknowledging that the underlying mechanisms were not directly assessed.

### 4.3. The Technical Performance Indicators in Elite Athletes Practicing Kata

The performance index derived from expert evaluations increased significantly in the BFB group, whereas no meaningful change was observed in the control condition. This pattern suggests that HRV-BFB may be associated with improvements in the technical execution of kata routines. Earlier work has proposed that biofeedback-based psychophysiological training can support performance optimization in athletes [41], although the specific pathways underlying such effects remain incompletely understood. One possibility discussed in the literature is that improved psychophysiological readiness may facilitate more consistent motor expression during skill execution. This interpretation is broadly compatible with previous findings linking physical preparedness to the quality of technical performance, as correlations have been reported between fitness-related attributes (e.g., strength, power) and precision of execution in martial arts [42].

In this context, the enhanced performance observed in the BFB group may reflect more stable attentional engagement and finer control of movement timing during kata routines. However, these explanations remain tentative, as the present study did not directly assess the cognitive or neuromuscular mechanisms underlying the performance changes.

### 4.4. Limitations and Future Directions

This study has several limitations that should be taken into account when interpreting the results. First, the sample size was modest, and although both groups were comparable in training experience, athletes represented different martial arts disciplines. This heterogeneity may have contributed to variability in technical demands and physiological responses. Second, the intervention consisted of a hybrid protocol with a relatively short overall duration; therefore, conclusions regarding the stability or long-term persistence of the observed changes should be made with caution. Third, although daily home practice was prescribed, adherence was monitored only through self-report rather than objective device-based logs, which may have introduced uncertainty regarding the actual training dose. Fourth, HRV indices were derived from photoplethysmography (PPG) rather than ECG, which is considered the gold standard. Although validated for short-term recordings, PPG-based HRV may be more sensitive to motion and peripheral factors and should therefore be interpreted with appropriate caution. Fifth, although expert scoring provided an ecologically valid measure of kata execution, performance ratings inevitably retain a subjective component.

Finally, this study did not include neurophysiological or psychological control measures (e.g., EEG, attentional or mood assessments), which limits the ability to determine the mechanisms linking autonomic regulation, sensorimotor adjustments, and performance outcomes. Future research with larger and more homogeneous samples, extended follow-up periods, objective monitoring of home practice, and multimodal physiological assessment is needed to confirm and expand upon these findings.

## 5. Conclusions

This study examined the short-term effects of a hybrid HRV-BFB protocol in elite martial arts athletes and identified several group-specific differences across autonomic, sensorimotor, and performance-related measures. Athletes who completed HRV-BFB demonstrated an improvement in expert-rated kata execution compared with a time-matched passive relaxation condition. The BFB group also showed a reduction in movement oscillation frequency and a trend toward faster choice reaction time, indicating potential benefits for neuromotor steadiness and cognitive–motor processing. These changes should be interpreted cautiously, as the underlying physiological or cognitive mechanisms were not directly assessed.

Passive relaxation, in contrast, was associated with short-term autonomic shifts without corresponding improvements in sensorimotor or performance outcomes. Taken together, the findings suggest that HRV-BFB may hold promise as a complementary approach for supporting technical performance in complex motor skills; however, the effects observed here are preliminary and short-term.

Future studies using larger and more homogeneous samples, objective monitoring of training adherence, longer intervention periods, and multimodal physiological and psychological assessments are needed to clarify the robustness of these effects and the processes through which HRV-BFB may influence motor performance.

## Figures and Tables

**Table 1:** Demographic and baseline characteristics of the participants.

S. No.	Parameters	Control Group (n = 20)	BFB Group (n = 20)	*p*-Value
1.	Age (years)	19.00 ± 1.75	20.25 ± 2.34	0.063
2.	Height (cm)	174.60 ± 7.76	174.15 ± 9.20	0.868
3.	Weight (kg)	65.28 ± 8.04	65.67 ± 11.51	0.899
4.	Sport experience (years)	11.47 ± 3.68	12.12 ± 3.68	0.580
5.	Females (n)	7	8	1.000
6.	Males (n)	13	12	1.000
7.	HR (bpm)	74.70 ± 13.09	73.79 ± 14.97	0.238

Notes: BFB—biofeedback. Data are presented as mean ± SD. HR—heart rate. *p* < 0.05 was considered statistically significant. Intergroup comparison was done using an unpaired *t*-test. Intergroup comparison was done using an unpaired chi-squared test (difference in sex distribution).

**Table 2:** Sport type distribution by group.

Sport Type	Control Group (n = 20)	BFB Group (n = 20)
Karate	10	10
Kyokushinkai	4	3
Taekwondo	1	4
Wushu	5	3
*p*-value: 0.251		

Notes: Intergroup comparison was done using an unpaired chi-squared test. *p* < 0.05 was considered statistically significant.

**Table 3:** Comparison of HRV, sensorimotor, and psychological parameters before and after intervention in the BFB and control groups (Me (IQR).

Parameters	Control (n = 20)		*p*	BFB (n = 20)		*p*
	Pre	Post		Pre	Post	
HRV parameters						
BVP amplitude mean, rel.	11.15 (8.55)	15.41 (5.18)	0.0365 **	11.9 (8.85)	13.1 (14.30)	0.3702
VLF, %	1.70 (1.76)	4.27(3.81)	0.0438 **	2.35 (2.54)	3.02 (5.54)	0.2322
LF, %	40.28 (49.24)	63.41(3.81)	0.0169 **	44.26 (39.86)	47.93 (33.80)	0.6274
HF, %	58.10 (50.37)	32.81(34.10)	0.0080 **	51.38 (37.00)	40.25 (42.52)	0.8812
LF, n.u.	40.93 (51.55)	65.58 (35.18)	0.0090 **	46.19 (39.66)	55.08 (40.28)	1.0000
HF, n.u.	59.02 (51.60)	34.41(35.00)	0.0090 **	53.80 (39.24)	44.91 (40.40)	1.0000
LF/HF ratio	0.69 (2.71)	1.90 (3.62)	0.0333 **	0.72 (2.41)	1.23 (2.25)	0.4688
Sensorimotor and psychological parameters						
Movement oscillation frequency, Hz	1.75 (1.00)	1.5 (1.20)	0.5861	1.5 (0.09)	1.1 (0.60)	0.0009 **
Choice reaction time, ms	311.28 (43.50)	316.97 (49.32)	0.8405	341.69 (70.46)	329.45 (72.86)	0.0793 *
Cognitive anxiety, score	15.00 (6.50)	15.00 (6.50)	0.5509	19.50 (9.50)	17.50 (8.50)	0.5286
Somatic anxiety, score	14.00 (5.00)	13.50 (4.50)	0.1158	15.50 (5.00)	16.00 (5.50)	0.8339
Self-confidence, score	30.00 (8.00)	30.00 (7.00)	0.2787	25.50 (6.50)	26.50 (9.50)	0.4838
Judges’ scores of kata performance						
Kata’s change score, % of max	0.00 (0.92)		-	0.43 (4.00)		0.0205 ^#^

Notes: * The differences between the parameters are significant (*p* < 0.1), ** the differences between the parameters are significant (*p* < 0.05), according to Wilcoxon signed-rank test; ^#^ based on the Mann–Whitney U test. Type of groups is used as the covariate. IQR—interquartile range.

## Data Availability

The data presented in this study are available on request from the corresponding author due to participant privacy and ethical restrictions.

## Supplementary Materials

The following supporting information can be downloaded at: https://www.mdpi.com/article/10.3390/sports14020051/s1, Table S1: Published reliability indices (ICC) and typical error estimates for standardized computerized sensorimotor tests corresponding to the tasks used in the present study.

## References

[1] Molinaro L., Taborri J., Montecchiani M., Rossi S. (2020). Assessing the Effects of Kata and Kumite Techniques on Physical Performance in Elite Karatekas. Sensors.

[2] Gökdere F., Uylas E., Çatıkkaş F., Günay E., Ceylan H., Ozgoren M. (2025). Integrating Kata Training into School Education: Effects on Sustained Attention and Cognitive Performance in 8–9-Year-Old Children. Children.

[3] Shaffer F., Ginsberg J.P. (2017). An Overview of Heart Rate Variability Metrics and Norms. Front. Public Health.

[4] Lehrer P.M., Gevirtz R. (2014). Heart rate variability biofeedback: How and why does it work?. Front. Psychol..

[5] Lehrer P., Kaur K., Sharma A., Shah K., Huseby R., Bhavsar J., Sgobba P., Zhang Y. (2020). Heart Rate Variability Biofeedback Improves Emotional and Physical Health and Performance: A Systematic Review and Meta Analysis. Appl. Psychophysiol. Biofeedback.

[6] Laborde S., Mosley E., Thayer J.F. (2017). Heart Rate Variability and Cardiac Vagal Tone in Psychophysiological Research—Recommendations for Experiment Planning, Data Analysis, and Data Reporting. Front. Psychol..

[7] Hirten R.P., Danieletto M., Landell K., Zweig M., Golden E., Pyzik R., Kaur S., Chang H., Helmus D., Sands B.E. (2024). Remote Short Sessions of Heart Rate Variability Biofeedback Monitored with Wearable Technology: Open-Label Prospective Feasibility Study. JMIR Ment. Health.

[8] Goessl V.C., Curtiss J.E., Hofmann S.G. (2017). The effect of heart rate variability biofeedback training on stress and anxiety: A meta-analysis. Psychol. Med..

[9] Paul M., Garg K. (2012). The effect of heart rate variability biofeedback on performance psychology of basketball players. Appl. Psychophysiol. Biofeedback.

[10] Pagaduan J., Chen Y.S., Fell J., Wu S.S.X. (2020). Can Heart Rate Variability Biofeedback Improve Athletic Performance? A Systematic Review. J. Hum. Kinet..

[11] Deschodt-Arsac V., Lalanne R., Spiluttini B., Bertin C., Arsac L. (2018). Effects of heart rate variability biofeedback training in athletes exposed to stress of university examinations. PLoS ONE.

[12] Li Q., Steward C.J., Cullen T., Che K., Zhou Y. (2022). Presleep Heart-Rate Variability Biofeedback Improves Mood and Sleep Quality in Chinese Winter Olympic Bobsleigh Athletes. Int. J. Sports Physiol. Perform..

[13] Herhaus B., Kalin A., Gouveris H., Petrowski K. (2022). Mobile Heart Rate Variability Biofeedback Improves Autonomic Activation and Subjective Sleep Quality of Healthy Adults—A Pilot Study. Front. Physiol..

[14] Yılmaz E., Aktop A., Abdioğlu A., Melekoğlu T., Nalbant M. (2025). The Effect of Heart Rate Variability Biofeedback on Recovery After Aerobic Exercise. Appl. Psychophysiol. Biofeedback.

[15] Porges S.W. (2007). The polyvagal perspective. Biol. Psychol..

[16] Bochaver K., Dovzhik L., Bondarev D. (2023). Psychological Diagnostics in Sport.

[17] Ferreira S., Raimundo A., del Pozo-Cruz J., Marmeleira J. (2021). Psychometric properties of computerized and hand-reaction time tests in older adults using long-term facilities with and without mild cognitive impairment. Exp. Gerontol..

[18] Deary I.J., Liewald D., Nissan J. (2011). A free, easy-to-use computer-based simple and four-choice reaction time programme: The Deary–Liewald reaction time task. Behav. Res. Methods.

[19] Sheridan S., Flowers K., Hursh K. (1986). Reliability of the Bassin Anticipation Timer. Percept. Mot. Skills.

[20] Stark T., Walker B., Phillips J.K., Fejer R., Beck R. (2011). Hand-held dynamometry correlation with the gold-standard isokinetic dynamometry: A systematic review. PM&R.

[21] Schuhfried GmbH (2011). Vienna Test System: Motor Performance Series (MLS) Test Manual.

[22] Ong N.C.H. (2015). The use of the Vienna Test System in sport psychology research: A review. Int. Rev. Sport Exerc. Psychol..

[23] Schäfer A., Vagedes J. (2013). How accurate is pulse rate variability as an estimate of heart rate variability? A review on studies comparing photoplethysmographic technology with an electrocardiogram. Int. J. Cardiol..

[24] Lehrer P. (2013). How Does Heart Rate Variability Biofeedback Work? Resonance, the Baroreflex, and Other Mechanisms. Biofeedback.

[25] Lehrer P.M., Vaschillo E., Vaschillo B. (2000). Resonant frequency biofeedback training to increase cardiac variability: Rationale and manual for training. Appl. Psychophysiol. Biofeedback.

[26] Berntson G.G., Bigger J.T., Eckberg D.L., Grossman P., Kaufmann P.G., Malik M., Nagaraja H.N., Porges S.W., Saul J.P., Stone P.H. (1997). Heart rate variability: Origins, methods, and interpretive caveats. Psychophysiology.

[27] Reyes del Paso G.A., Langewitz W., Mulder L.J., van Roon A., Duschek S. (2013). The utility of low frequency heart rate variability as an index of sympathetic cardiac tone: A review with emphasis on a reanalysis of previous studies. Psychophysiology.

[28] Lin I., Fan S., Yen C., Yeh Y., Tang T., Huang M., Liu T., Wang P., Lin H., Tsai H. (2019). Heart Rate Variability Biofeedback Increased Autonomic Activation and Improved Symptoms of Depression and Insomnia among Patients with Major Depression Disorder. Clin. Psychopharmacol. Neurosci..

[29] Jung H., Yoo H.J., Choi P., Nashiro K., Min J., Cho C., Thayer J.F., Lehrer P., Mather M. (2025). Changes in Negative Emotions Across Five Weeks of HRV Biofeedback Intervention were Mediated by Changes in Resting Heart Rate Variability. Appl. Psychophysiol. Biofeedback.

[30] Thayer J.F., Lane R.D. (2009). Claude Bernard and the heart-brain connection: Further elaboration of a model of neurovisceral integration. Neurosci. Biobehav. Rev..

[31] Mather M., Thayer J.F. (2018). How heart rate variability affects emotion regulation brain networks. Curr. Opin. Behav. Sci..

[32] Hansen A.L., Johnsen B.H., Thayer J.F. (2003). Vagal influence on working memory and attention. Int. J. Psychophysiol..

[33] Park G., Thayer J.F. (2014). From the heart to the mind: Cardiac vagal tone modulates top-down and bottom-up visual perception and attention to emotional stimuli. Front. Psychol..

[34] Prinsloo G.E., Rauch H.G., Karpul D., Derman W.E. (2013). The effect of a single session of short duration heart rate variability biofeedback on EEG: A pilot study. Appl. Psychophysiol. Biofeedback.

[35] Yoo H.J., Nashiro K., Min J., Cho C., Bachman S.L., Nasseri P., Porat S., Dutt S., Grigoryan V., Choi P. (2022). Heart rate variability (HRV) changes and cortical volume changes in a randomized trial of five weeks of daily HRV biofeedback in younger and older adults. Int. J. Psychophysiol..

[36] Layton C. (1993). Reaction + movement-time and sidedness in Shotokan karate students. Percept. Mot. Ski..

[37] Mori S., Ohtani Y., Imanaka K. (2002). Reaction times and anticipatory skills of karate athletes. Hum. Mov. Sci..

[38] Fontani G., Lodi L., Felici A., Migliorini S., Corradeschi F. (2006). Attention in athletes of high and low experience engaged in different open skill sports. Percept. Mot. Ski..

[39] Williams A.M., Elliott D. (1999). Anxiety, Expertise, and Visual Search Strategy in Karate. J. Sport Exerc. Psychol..

[40] Goethel M.F., Vilas-Boas J.P., Machado L., Ervilha U.F., Moreira P.V.S., Bendilatti A.R., Hamill J., Cardozo A.C., Gonçalves M. (2023). Performance, Perceptual and Reaction Skills and Neuromuscular Control Indicators of High-Level Karate Athletes in the Execution of the Gyaku Tsuki Punch. Biomechanics.

[41] Jiménez Morgan S., Molina Mora J.A. (2017). Effect of Heart Rate Variability Biofeedback on Sport Performance, a Systematic Review. Appl. Psychophysiol. Biofeedback.

[42] Ribas M.R., Pereira M.A.S., Barbosa T.A., Lass A.D., Bassan J.C. (2020). Tactical and technical performance analysis of the male 65 kg category at the brazilian shotokan karate championship. J. Phys. Educ..

