# Supplementary material for: One-Week Home-Based HRV Biofeedback with Supervised Sessions Versus Passive Relaxation: Effects on Autonomic, Sensorimotor Functions and Kata Performance in Eastern Martial Arts Athletes
Source: Sports (Basel). 2026 Feb 3;14(2):51. doi: 10.3390/sports14020051 (PMC12944102; doi:10.3390/sports14020051)
Supplement: Supplementary file 1 [file sports-14-00051-s001.zip › sports-4093165-supplementary.pdf]

Supplementary Table S1. Published reliability indices (ICC) and typical error estimates for standardized computerized sensorimotor tests corresponding to the tasks used in the present study.

Test	Main outcome	Published reliability (ICC)	Typical Error	Source
Reaction Time	Choice and simple RT latency	0.61 (SRT) – moderate<br>0.89 (CRT) - high	18–25 ms (SRT)<br>25–35 ms (CRT)	Ferreira, S et al., 2021 [1],<br>Deary IJ, Liewald D, Nissan J, 2011 [2];
Coincidence Anticipation Timing Test	RT, anticipation	0.88 (across sports)	15–25 ms	Sheridan, S et al., 1986 [3];
Dynamometry	force accuracy	0.80–0.98	3–12% (across 32 studies)	Stark T et al., 2011 [4];
Stylus Tracking Task	Number of errors,<br>Contact time	0.80–0.92	1–3 errors (5–15%)	Schuhfried GmbH. Vienna Test System 2011 [5];<br>Ong, N. C. H. 2015 [6];

Notes: SRT – simple reaction time, CRT – choice reaction time, ICC - intraclass correlation coefficient

References:

1. Ferreira S, Raimundo A, del Pozo-Cruz J, Marmeleira J. Psychometric properties of computerized and hand-reaction time tests in older adults using long-term facilities with and without mild cognitive impairment. Exp Gerontol. 2021;147:111271. doi:10.1016/j.exger.2021.111271
2. Deary IJ, Liewald D, Nissan J. A free, easy-to-use computer-based simple and four-choice reaction time programme: the Deary–Liewald reaction time task. Behav Res Methods. 2011;43(1):258–268. doi:10.3758/s13428-010-0024-1
3. Sheridan S, Flowers K, Hursh K. Reliability of the Bassin Anticipation Timer. Percept Mot Skills. 1986;62(1):55–60.
4. Stark T, Walker B, Phillips JK, Fejer R, Beck R. Hand-held dynamometry correlation with the gold-standard isokinetic dynamometry: a systematic review. PM&R. 2011;3(5):472–479. doi:10.1016/j.pmrj.2010.10.025
5. Schuhfried GmbH. Vienna Test System: Motor Performance Series (MLS) Test Manual. Mödling, Austria: Schuhfried GmbH; 2011.
6. Ong NCH. The use of the Vienna Test System in sport psychology research: a review. Int Rev Sport Exerc Psychol. 2015;8(1):1–20. doi:10.1080/1750984X.2015.1061581
